# A qualitative analysis of experiences in veteran voices and visions groups: developing a participant-informed model of connection, acceptance, and social integration

**DOI:** 10.3389/fpsyt.2026.1717311

**Published:** 2026-09-04

**Authors:** LeeAnn Akouri-Shan, Logan Leathem, Erica Hua Fletcher, Ronald Calderon, Susanna Friedlander, Ippolytos Kalofonos

**Affiliations:** 1 Department of Psychiatry and Biobehavioral Sciences, Semel Institute for Neuroscience and Human Behavior, University of Los Angeles, California, Los Angeles, CA, United States; 2 Veterans Affairs Greater Los Angeles Healthcare System, Los Angeles, CA, United States

**Keywords:** conceptual model, hallucinations, hearing voices, support group, veteran mental health, peer support, recovery, qualitative research

## Abstract

**Purpose/Aims:**

This study applied a model of transformation for Hearing Voices Groups in the US developed by Hornstein et al. to Veteran Voices and Visions (VVV) groups adapted for a Veterans Affairs healthcare system. The aims were twofold: (1) identify instances of Discovery, Reframing, and Change and empirically confirm the salience of this model for our groups; and (2) identify the specific dynamics of change in our groups, thus refining and elaborating upon the existing model.

**Method:**

This study used a qualitative design with in-depth semi-structured interviews that were assessed pre-intervention, at 8 weeks, and at 16 weeks with 24 participants within five groups. Interviews were recorded and transcribed. Abductive analysis of the interview transcripts was conducted, first drawing theoretical insights from Hornstein et al.’s framework to organize and synthesize qualitative data deductively then using inductive rapid qualitative analysis to enrich and develop a theory of change specific to this study population.

**Findings:**

While data from this study mostly align with the Hornstein et al. model of transformation, in our data, findings from each phase seemed to emphasize a different theme than that foregrounded in the Hornstein et al. model. We articulate these differences in a refined model of transformation. Our model makes an additional refinement by delineating two distinct tracks: an inner track reflecting how the group influences participants’ relationships with themselves and their own experiences, and an outer track reflecting how participants relate to fellow group members. We also consider barriers and facilitators within each phase.

**Implications:**

Our findings indicate that the Hearing Voices approach can be successfully adapted to a large public mental healthcare system. They also provide a model for engagement with groups during the initial weeks and months of group attendance. VVV groups provided a valuable setting for connection, acceptance, and community that led to a greater sense of belonging and social integration.

## Introduction

### Clinical approaches to psychosis

Psychotic experiences vary widely, often involving unusual perceptions – such as hearing voices or seeing visions – or beliefs not typically shared by others. Clinical services traditionally position psychosis as a marker of pathology and are oriented toward eliminating, reducing, suppressing, or otherwise finding ways to correct what are understood to be distorted perceptions of reality (1–4). First person accounts of psychosis, on the other hand, reveal phenomenologically rich, nuanced experiences relating to sense of self and one’s place in the world and a complex range of associated emotions (5, 6). While reduction and suppression of psychosis can be helpful, particularly in times of crisis, a health system that is organized around prioritizing this approach risks a reduction and flattening of subjectivity that can silence and exclude human experience, foreclosing opportunities for meaning and growth and rendering the person experiencing psychosis powerless and untrustworthy in the context of their own care and recovery (7, 8). Over the past forty years, service users have called for a person-centered approach to mental health care that is attuned to the process of strengthening resilience in the existential domain of personal recovery, fostering meaning, identity, and sense of purpose, as well as in the social domain, promoting citizenship, connectedness, and social resources (2, 9–12). This has contributed to calls for the concept of personal recovery, rather than symptom remission, to serve as the organizing and integrating principle for mental health services (2, 13, 14).

These calls have been taken up institutionally for over twenty years. In the United States, the President’s New Freedom Commission report of 2002 (15) stated “care must focus on increasing patients’ ability to successfully cope with life’s challenges, on facilitating recovery, and on building resilience, not just on managing symptoms.” The Veterans Affairs Healthcare System (VA), one of the largest public mental health providers in the country, adopted this orientation in 2004 (16). Yet, studies and evaluations of VA and other health systems since that time indicate that treatment remains driven by symptom reduction for diagnosable disorders through application of evidence-based practices, and there remains substantial room to grow in a recovery-oriented, person-centered direction (2, 17, 18).

This study is a part of a project that seeks to facilitate the transformation of the VA to be a more recovery-oriented health system by adapting Hearing Voices (HV), a meaning-centered, recovery-oriented, peer-led approach to psychosis, to the VA Greater Los Angeles Healthcare System (VAGLAHS), which serves a large and diverse community of Veterans in the Southern California region of the US.

### The Hearing Voices approach

The Hearing Voices (HV) approach emerged from a partnership between a voice hearer, her psychiatrist, and a researcher in the Netherlands in the late 1980s (19). It has developed into the world-wide Hearing Voices Movement (HVM), a survivor-led initiative advocating for the reform of mental health services, promoting the rights of voice hearers, and collaborating with individuals to explore the meaning of their experiences and work toward recovery (20).

The HV approach takes a non-pathologizing, non-judgmental approach to psychosis, understanding these experiences as a natural part of being human and accepting diverse explanations for the origins of voices (20). It draws on the strengths and unique perspectives of voice hearers to better understand how they relate to and cope with voices and related phenomena (21). The approach advocates for a holistic, empowering understanding of these experiences, emphasizing the cultural, contextual, and personal factors that shape them (19, 20).

### Hearing voices groups

The core of the HVM is organizing self-help groups run by and for people who hear voices and have other unusual experiences that often get diagnosed as psychosis (20). HV groups (HVGs) are typically co-facilitated by a pair of facilitators, at least one of whom should be a voice hearer. The groups focus on normalizing unusual perceptual experiences, encouraging the process of meaning making by considering diverse explanatory frameworks, sharing strategies for coping and acceptance, and building community among voice hearers (20).

HVGs have historically operated independently from clinical services, validating subjective understandings of voice hearing, and emphasizing expertise-by-experience and learning to live with voices (20, 22, 23). HVGs are distinct from clinical services in that the goal is to build mutual relationships and supportive community, not to achieve a pre-specified clinical outcome (24, 25). The HVM prioritizes personal experience and testimony as a primary source of evidence (20). Due to these differences, there have been questions whether HVGs could operate within clinical services (20, 26–29). As such, the HVM has had an “uneasy” relationship with the traditional research methods of the medical and social sciences, which tend to favor clinical outcomes over personal experiences of transformation (20). Nonetheless, there is a tradition of research and collaboration between the HVM and academic and clinical allies, and this study is positioned within that literature (22, 27, 30–33). As the popularity of the HVM grows, a greater understanding of how HVGs work, as well as factors that facilitate and impede change, is essential for strengthening their impact and enhancing the lives of those who participate in them.

### Hornstein et al.’s three-phase model of transformation

Drawing from extensive group facilitation experiences as well as qualitative study of HV participants in the US, Hornstein et al. (24) proposed a three-phase model of transformation to illustrate how change occurs within a HV group. The model emphasizes a non-linear, evolving process of personal growth, where group members move between phases of Discovery, Reframing, and Change. In the Discovery phase, group members come to learn that others share experiences like theirs but may understand or respond to their experiences differently. Further, they come to view the group as a safe space where such experiences can be discussed openly and without consequences. In the Reframing phase, group members begin to experiment with new ways of understanding and living with their experiences, particularly in relation to their unique life circumstances. They may negotiate new relationships with their voices, find that voices themselves may change, and develop greater confidence and self-efficacy from new ways of relating to voices. In the Change phase, group members take new chances outside the group and address valued life projects, such as reconnecting with family or applying for jobs, and use group support to deal with setbacks.

### The current study

The current study is embedded within a larger project adapting Hearing Voices groups to VAGLAHS that involves collaboration among VA researchers, clinicians, peer specialists, and service users, and US-based Hearing Voices leaders. The groups, called Veteran Voices and Visions (VVV), are conducted virtually, run within the VA, and designed to conform to HV practices and values. In this study, our aims were twofold: (1) identify instances of Discovery, Reframing, and Change, as proposed by the Hornstein et al. (24) model, and empirically confirm the salience of this model for our VVV groups; and (2) refine and elaborate upon the Hornstein et al. (24) model by identifying specific dynamics of change in our groups.

Quantitative data and a focused portion of the qualitative data from this study were analyzed using a modified grounded theory approach and previously published (34). Here, we focus exclusively on our qualitative data, utilizing the Hornstein et al. (24) model as the point of departure to examine Veteran experiences in VVV groups and explore whether these groups had similar dynamics to the community-based, mostly in-person HV groups that Hornstein et al. (24) studied.

## Methods

### Study design

To understand participant experiences in our VVV groups, we conducted an abductive rapid qualitative analysis of semi-structured interviews with participants pre-intervention, at 8 weeks, and at 16 weeks, based on the Hornstein et al. (24) framework. We received human subjects approval from the VAGLAHS institutional review board.

### Setting

VAGLAHS serves a large and diverse community of Veterans in the Southern California region of the US. It cares for several thousand Veterans with serious mental illness diagnoses, providing a range of services including housing support, intensive case management, recovery and rehabilitative services (e.g., vocational rehabilitation), psychiatric consultation (e.g., medication management), a range of psychotherapies (e.g., individual and group psychotherapy), substance abuse services, and emergency and inpatient services when needed. VA is a nationwide healthcare system and is the largest employer of peer support specialists in the country.

### Intervention

A VA peer support specialist and VA mental health clinician dyad co-facilitated the VVV groups. Due to the COVID-19 pandemic, all groups were held virtually. Groups met online for one hour per week for 16 weeks. All participants were asked about their comfort with participating in a virtual group during pre-intervention and follow-up interviews. Participants were also informed that camera use during group was optional and that they could still participate with their cameras off if desired. All facilitators received specialized training from the Wildflower Alliance, a peer support and advocacy organization that is a leader in US-based Hearing Voices group facilitation training (35). In accordance with HV values, Wildflower Alliance trained facilitators to explore diverse participant experiences through multiple frameworks of understanding, with a focus on personal meaning. Topics such as predatory recruitment, reintegration into civilian life, harassment, bullying, sexual trauma experienced in the military, and difficulties dealing with government bureaucracies are centered in the HV training developed for VVV groups, emphasizing life experiences unique to Veterans. Future VVV facilitators also practiced establishing a safe space where any topic, including frustrations with medical and mental health care, could be openly discussed. Facilitators met together twice monthly to promote fidelity and discuss challenges or concerns.

### Recruitment

Group participants were referred by VA mental health clinicians and were recruited from multiple clinical programs across VAGLAHS, including outpatient psychiatry and psychology clinics, recovery-oriented group programs, residential rehabilitation programs, substance use programs, case managers, peer specialists, housing specialists, and inpatient programs. Eligibility criteria included: active, chronic psychosis of at least 6 months duration not exclusively due to substance use and ability to attend virtual weekly group sessions as an outpatient. All VVV groups were begun for the study, and all participants were new to the groups and the HV approach. In total, 29 Veterans consented to the study, attended at least one group, and provided at least one interview. Interviews from 24 Veterans who attended at least one group and completed pre-intervention and 8-week and/or 16-week interviews are included in this analysis (20 completed all 3 interviews, 4 completed only pre-intervention and 8-week interviews).

### Sample characteristics

Demographic and clinical characteristics of the sample are presented in **Table 1**. Gender, race/ethnicity, and marital status were self-reported, and other data were obtained via chart review. The 24 Veterans in this study ranged in age from 25 to 61 years old (*M* = 41.9, *SD* = 11.3). Most participants were male (79.1%), never married (70.8%), and had completed at least a high school education (95.7%). The sample was racially and ethnically diverse, with half identifying as Black/African American (50%), followed by Latine/Hispanic (29.2%), White (16.7%), and Asian, Native Hawaiian, or Pacific Islander (4.1%). All participants were diagnosed with a psychosis spectrum disorder and were prescribed antipsychotic medication, with many also diagnosed with comorbid psychiatric concerns. Relatively few Veterans had current substance use disorder diagnoses. While 11 Veterans (46%) currently used cannabis, only 2 (8%) had an active cannabis use disorder while 5 (21%) had a cannabis use disorder in remission, perhaps reflecting the recent legalization and normalization of cannabis use in California. Similarly, 10 Veterans (42%) used alcohol, but only 2 (8%) had an alcohol use disorder, while 12 (50%) had a history of alcohol abuse but were in remission. There were higher rates of substance use disorders in remission, perhaps reflecting our recruitment strategy that included substance use clinics and rehabilitation programs. Most participants (83%) reported at least one prior psychiatric hospitalization. On average, Veterans attended 10 of the 16 group sessions. Veterans in the study received their usual care through VAGLAHS.

**Table 1:** Sample characteristics.

Characteristic	N(%)
Age (Years; Range = 25–61)	
*M*, *SD*	41.9, 11.3
Gender	
Male	19 (79%)
Female	5 (21%)
Race/Ethnicity	
Asian, Native Hawaiian, Pacific Islander	1 (4%)
Black/African American	12 (50%)
Latine/Hispanic	7 (29%)
White	4 (17%)
Highest Level of Education	
Some high school	1 (4%)
High school graduate/GED	10 (42%)
Some college	12 (50%)
College graduate	1 (4%)
Marital Status	
Never married	17 (71%)
Married	1 (4%)
Divorced/separated	4 (17%)
Unknown	2 (8%)
Diagnosis	
Psychosis spectrum	24 (100%)
PTSD	8 (33%)
Mood disorder	7 (26%)
Anxiety disorder	4 (17%)
Substance Use	
Cannabis Use	11 (46%)
Cannabis Use Disorder	2 (8%)
Cannabis Use Disorder, in remission	5 (21%)
Alcohol Use	10 (42%)
Alcohol Use Disorder	2 (8%)
Alcohol Use Disorder, in remission	12 (50%)
Methamphetamine Use	1 (4%)
Methamphetamine Use Disorder	1 (4%)
Methamphetamine Use Disorder, in remission	4 (17%)
Cocaine Use	0
Cocaine Use Disorder	0
Cocaine Use Disorder, in remission	9 (38%)
Opiate Use	0
Opiate Use Disorder	0
Opiate Use Disorder, in remission	3 (13%)
Lifetime Number of Psychiatric Hospitalizations (Range = 0–10)	
*M, SD*	3.0, 3.0
≥ 1	20 (83%)
Prescribed Antipsychotics	
	24 (100%)
Group Attendance (Number of Sessions; Range = 1–16)	
*M, SD*	10, 4.8

Diagnosis numbers exceed 100% due to most Veterans carrying multiple diagnoses.

### Data collection

Data collection for this study took place from February to December 2021. Interviews were conducted prior to the participant’s first group, at 8 weeks, and again at 16 weeks. Our semi-structured interview guide explored overall experiences in the group, changes in one’s understanding of their experiences and ability to live with them, and how involvement in the group impacted relationships and life outside the group (see Appendix 1). Interviews were audio recorded and professionally transcribed.

Two interviewers performed all the qualitative interviews virtually (IK and RC). IK is an MD-PhD psychiatrist-medical anthropologist who practices at VAGLAHS. He is the principal investigator of the study and co-facilitated at least one session of four of the five VVV groups in this study but was the regular facilitator in only one of the groups. He has co-facilitated VVV groups outside of the study and HV groups outside of the VA. He worked at an outpatient clinic where four of the participants were patients during the study, though he did not have relationships with any of the Veteran participants prior to the start of the study. He conducted all the pre-intervention interviews and a portion of the 8-week and 16-week interviews. RC is a social worker and health services researcher who conducted most of the 8-week and 16-week interviews. He also practices clinically at VAGLAHS but had no pre-existing relationships with study participants. Interviewers shared their occupation and background with study participants. Interviews averaged 45 minutes in length.

### Data analysis

We conducted abductive analysis (36) of the interview transcripts, first drawing theoretical insights from Hornstein et al. (24)’s framework to organize and synthesize qualitative data deductively, then using inductive rapid qualitative analysis (37) to enrich and develop a theory of change specific to our population. We chose an abductive approach to sequentially integrate deductive and inductive analyses. We used the rapid qualitative analytic approach described here to maximize efficiency and consistency across our research team. Two authors (LAS and LL) who were not involved in data collection or group facilitation independently reviewed all interview transcripts. They condensed and synthesized data from the interviews, transferring data from each interview into a templated summary based on the interview question domains. Interview themes were then discussed with members of the research team (LAS, LL, EF, SF, IK) to guide further analysis. From there, data regarding how Veterans engaged in the discovery process, reframed their experiences, or engaged in meaningful life projects – based on the Hornstein et al. (24) model – were extracted and organized in a thematic matrix by participant and time point, allowing for the analysis of trends across Veterans and changes within Veterans from pre-intervention to 8-week and to 16-week time points. Instances of confirming or disconfirming cases, based on the Hornstein et al. (24) model, as well as departures from the model, were noted.

**Figure 1:**
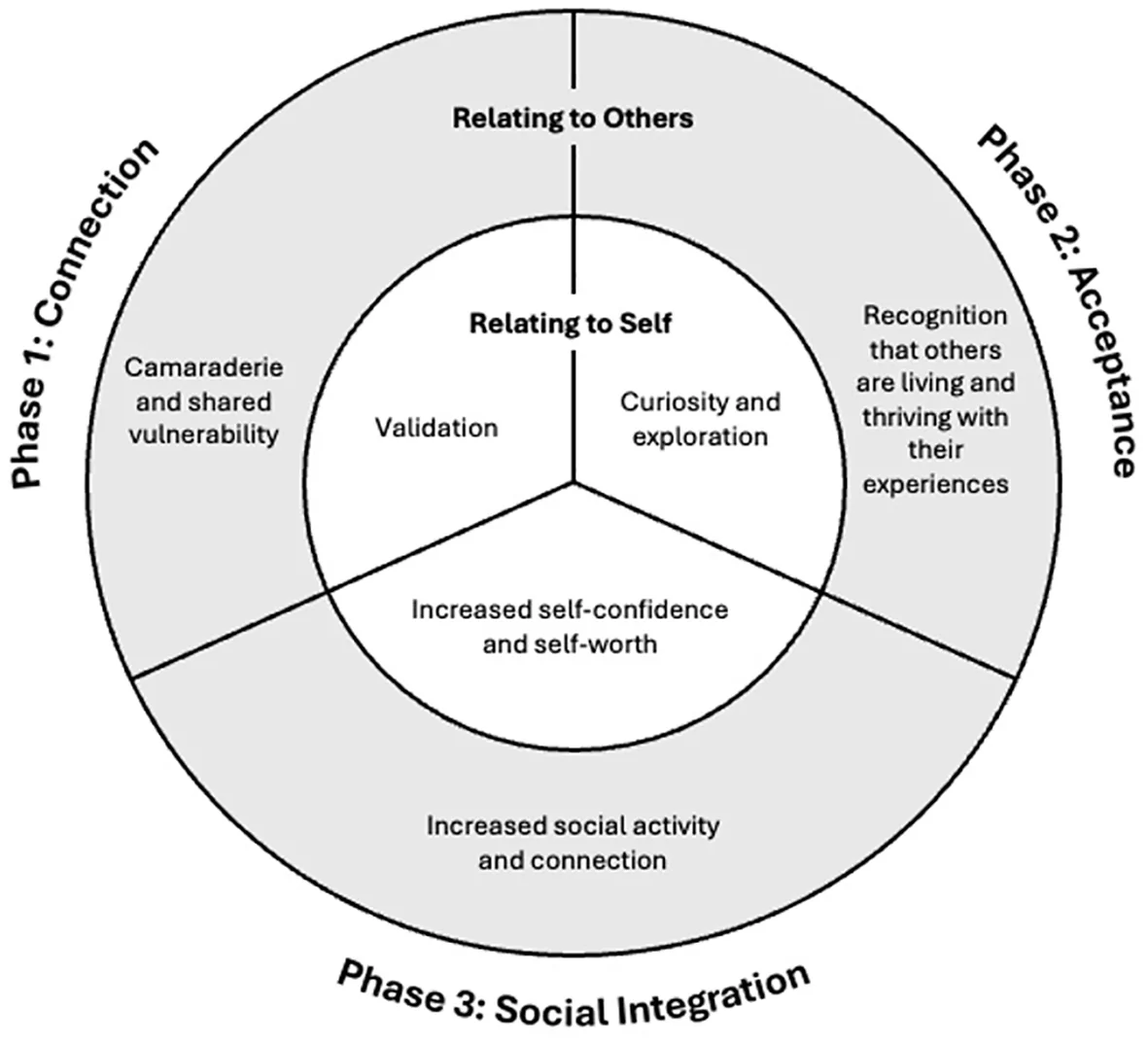
Proposed model of transformation in veteran voices and visions groups.

The research team then examined the data in the matrix and inductively reviewed it for emergent themes using a consensus approach to characterize dominant participant experiences with respect to the three phases. The identified themes were discussed with one of the study participants, and their feedback was incorporated into the refined conceptual model that resulted (**Figure 1**).

The repeated interviews allowed for the detection of changes that may have resulted from group involvement within individual participants. Pre-intervention interviews inquired about participant understandings of their experiences, how they live or cope with them, and their social relationships and supports. Eight- and 16-week interviews explored whether there were any changes in any of these domains following group participation, thus allowing us to assess the specific area of change for each individual participant.

## Results

This results section presents findings as they related to each phase of Hornstein et al. (24)’s model: Discovery, Reframing, and Change. Within each of these sections, both confirming and disconfirming evidence of Hornstein et al. (24)’s phases are provided. While data from this study mostly align with the Hornstein et al. (24) model of transformation, findings from each phase emphasized a different theme than that foregrounded by the Hornstein et al. (24) model. We articulate these differences in a refined model of transformation presented in **Figure 1**. Our refinements are noted in the section subtitles discussing each respective phase: From Discovery to Connection; From Reframing to Acceptance; and From Change to Social Integration. Our model makes an additional refinement by delineating two distinct tracks: an inner track reflecting how the group influences participants’ relationships with themselves and their own experiences, and an outer track reflecting how participants relate to fellow group members. The sections below elaborate on each phase of our proposed model, comparing it with Hornstein et al. (24)’s framework in the context of the current study findings, while also considering barriers and facilitators within each phase (**Table 2**). The qualitative data presented here reflect a heterogeneity of experiences, indicating that different individuals will experience the same groups in distinct ways.

**Table 2:** Facilitators and barriers of transformation across model phases.

Phase	Domain	Facilitators	Barriers
1. Connection	*Relating to Self*	- Validating, nonjudgmental group atmosphere - Open invitation to share - Others modeling vulnerability	- Holding certain military values – e.g., stoicism; deprioritization of self/needs
	*Relating to Others*	- Feeling of camaraderie and shared experiences - Flexible virtual format	- Not resonating with others’ stories - Not feeling heard - Receiving unwanted advice - Feeling pressure to share - Unequal participation from group members - Concerns about oversharing - Suspiciousness - Others keeping their cameras off - Preference for more structured interaction
2. Acceptance	*Relating to Self*	- Validation of experience-based expertise - Acceptance of multiple frameworks beyond illness model - Use of tools such as voice mapping and voice dialogue - Curiosity and respect for experiences - Self-compassion	- Fear of engaging with experiences - Belief that examining experiences is unsafe - Preference for avoidant coping strategies - Negative messages from prior treatment/society - Belief that further exploration of experiences is unnecessary/offers no benefit
	*Relating to Others*	- Curiosity - Respect for differences - Supportive feedback	- Fear of hearing others’ experiences - Feeling invalidated
3. Social Integration	*Relating to Self*	- Feeling a sense of agency or self-efficacy - Increased social motivation	- Feeling confined by illness/condition - Feeling incapable of taking risks
	*Relating to Others*	- Identifying with other group members; recognizing oneself in others’ stories - Seeing others take risks or initiate change - Feeling supported by the group	- Feeling content with – or resigned to – present conditions - Fear of distress and potential rejection

### From discovery to connection

In their proposed three-phase model of transformation, Hornstein et al. (24) describe the Discovery phase as an initial period of change in which group participants are exposed to a diverse range of voice hearing experiences (including differences in content, explanatory frameworks, coping styles, and ways of living with these experiences) in a nonjudgmental, person-centered context. The authors posit that this process can lead to an increased sense of awareness that is associated with increased feelings of “wonder and relief” (24, p. 6), belongingness, and hope, and decreased avoidance associated with voice hearing experiences.

In line with Hornstein et al. (24)’s model, most Veterans (17/24) in the current study reported feeling validated and less alone in their experiences because of participating in the group. These benefits were endorsed even among Veterans who reported minimal changes in other proposed phases of Hornstein et al. (24)’s model. One Veteran stated, “I’m not alone in my struggle. There is help if you seek it … Just listening to everybody else gives me hope” [17, interview 2]^1^. For some Veterans, the group setting was the first place where they realized others could have similar experiences. As one Veteran shared, “…for a long time, I just thought… ‘What’s wrong with me?’ and I thought it was just me” [29, interview 2]. Another Veteran compared the unique group process to his prior treatment experiences, noting, “I feel like I’m part of a team, rather than feeling like I’m under a microscope … other times I’ve met with psychologists and psychiatrists, and it feels like … they’re extracting data from you” [25, interview 2]. This Veteran also felt more hope after participating in the group, stating, “It’s nice to see a light at the end of the tunnel … this group has shown me that … even with what I’m going through, I can still … find a path to success and happiness.”

Several factors appeared to interfere with the process of discovery. One Veteran described perceived invalidation from other group members when discussing his interpretation of voices, stating, “Like they’re not even willing to listen to what I’m saying. I’ve shared videos and they don’t even want to respond or share” [2, interview 3]. Other Veterans noted discomfort from receiving unwanted or unhelpful advice, feeling pressured to share their experiences, and difficulty trusting other group members.

Some Veterans also highlighted other difficult group dynamics relating to the amount each participant shared. One Veteran expressed, “Sometimes a lot of people do all the talking, and I’m doing the listening” [18, interview 3]. Another Veteran described feeling self-conscious about talking too much and being perceived as a “know it all”, stating, “I wish other people would talk, too” [9, interview 2]. Similarly, one Veteran reported that inconsistent participation from other group members interfered with her ability to establish trust [10, interview 2]. A Veteran who initially reported a positive experience [20, interview 2] only attended the first group session and did not return for subsequent sessions. Later, she shared that she struggled to trust the facilitators and other group members [20, interview 3]. It was also noted that at-times smaller group sizes (e.g., 2–4 members) made meaningful engagement challenging, and that sometimes most of the group time was spent on “small talk” rather than deeper topics. As one Veteran stated, “Sometimes … it’s slow to start and then it gets going and then it’s done too fast” [25, interview 2]. While these observations may suggest a preference for deeper conversations over small talk, it should be noted that casual conversations may also serve a valuable role in HV groups, helping build rapport and foster feelings of comfort and connection. Finally, two Veterans did not endorse any changes related to Hornstein et al. (24)’s proposed Discovery phase. One of these Veterans did not identify as a voice hearer, whereas the other Veteran only attended the first group.

Based on these findings, it appears that the process of discovery as outlined by Hornstein et al. (24) may best describe Veterans in instances where there is: a) some personal acknowledgement of unusual experiences (regardless of explanatory framework), b) some minimum level of group participation, and c) a perceived solidarity and community in the group built around shared experiences.

Hornstein et al. (24)’s conceptualization of the Discovery phase, which focuses on the sharing of unusual experiences among group members, may not fully capture the broader benefits we identified with VVV groups. Several participants described how being part of the group provided a general sense of connection and community, particularly when mental health challenges resulted in a lack of outside support. After hearing about another group member overcoming personal struggles in securing better housing, one Veteran expressed, “We were all super happy for him and super proud that he found somewhere else [i.e., another place to live] that was safer and better for him” [30, interview 3]. Another Veteran emphasized that group members are genuinely invested in each other’s well-being, which helps foster a sense of accountability: “You got to show up, because other people are counting on you to show up. Like, if a person is missing from the group, I’d be like ‘where are they? Where is so-and-so? How come he ain’t on here? Or … where is she at? Is she sick? What’s going on?’” [21, interview 2]. Additionally, the opportunity to encourage others in the group was noted to be a healing experience. As one Veteran stated, “By helping [other group members], I’m helping myself” [9, interview 2].

For some participants, feeling validated and having the opportunity to connect with others in this unique context was a powerful experience, regardless of any additional discoveries or changes in behavior or functioning. The most common sentiment expressed by Veterans in our groups was the newfound sense of connection, of no longer being alone in their experiences. Many of the participants described a profound sense of alienation and isolation that was shattered by the group: Veterans were able to share about their experiences in the military and struggles with reintegration after their service ended and felt camaraderie with fellow Veterans living with unusual experiences. One participant favorably compared the groups to other groups he had experienced: “that fact that we have one shared experience [hearing voices] is something that makes me feel more comfortable. …I also feel more comfortable having the same people show up to group … and people remember what you say in group” [15, interview 2]. This Veteran noted the commonality of experience, the consistency of attendance, and the continuity of group members contributing to comforting relationships and camaraderie. They also resonated with what others shared: “one of the Veterans shared that they tend to hear voices when they’re stressed, angry, upset. That’s something I share with them.” Feedback from group members also appeared to serve as valuable information that could be used to challenge stigmatizing beliefs about unusual experiences.

### Facilitators and barriers to connection

The most important facilitator of connection was creating a space of nonjudgemental openness and collaboration in which all participants were invited to share their experiences; such experiences were then received and validated with nonjudgmental curiosity. Participants are regarded as the experts of their own experiences, a core HV value. The peer facilitators often played an important role in this process by sharing their own experiences and modeling vulnerability, but frequently group participants played this role as well by opening up about their own experiences. As previously mentioned, one likely facilitator to connection and cohesion was the camaraderie every participant shared as a Veteran. Another facilitator identified by several participants was the lack of pressure to share, and the fact that group participants speak more frequently than group facilitators. Likewise, a Veteran shared that they appreciated being able to just listen in early groups but then become comfortable sharing as the groups progressed [15, interview 3].

While some Veterans mentioned feeling more comfortable sharing personal experiences with other Veterans than with civilians, certain aspects of military culture – such as values of discipline (as mentioned by one Veteran [26, interview 2]), stoicism, and the deprioritization of individual needs – may act as barriers to group participation and to connection. Participants frequently described a military culture that discouraged help-seeking, framed help-seeking as a sign of weakness, and contributed to delays in their own search for help.

For a few Veterans, perceived invalidation seemed to impede connection. Veterans at times mentioned feeling invalidated by participants who seemed to doubt the veracity of their claims, offered unsolicited advice, or provided otherwise unwanted or unwelcome responses. Unequal participation from group members also appeared to interfere with group cohesion and trust. All groups were held online, which may have influenced Veterans’ level of participation or ability to read and respond to social cues. For example, one Veteran described discomfort interacting with participants who kept their cameras off during the group: “We can’t see everybody. I don’t think that’s fair” [21, interview 3]. However, only a few Veterans expressed preference for in-person versus virtual groups, while quite a few Veterans expressed preference for virtual groups. One Veteran stated, “I like [virtual groups] because it’s more flexible” [10, interview 3]. Other Veterans described feeling “less paranoid” when interacting with others in an online format [18, interview 2], as well as finding it “easier to open up” [25, interview 2]. One Veteran, when asked about in-person groups said, “Well, I’m not ready for that” [18, interview 3]. These findings highlight potential challenges in meeting the varying needs and preferences of Veterans in the group while ensuring a safe space for all to share.

Our findings also highlight the role of facilitators to set expectations around sharing in group and creating equal opportunities for group members to participate (if desired). A few Veterans in this study expressed a desire for facilitators to encourage sharing by calling on group members or to provide more structure by suggesting topics for discussion.

### From reframing to acceptance

According to Hornstein et al., (24) the reframing process involves learning new ways of relating to and coping with unusual experiences. This process can also include initial changes in the experiences themselves (e.g., reduced frequency/intensity of voices).

Many Veterans in the current study reported increased acceptance (“They’ll probably never go away, but they’re just something I have to learn to deal with” [18, interview 2]), an increased sense of self-control (“I can’t turn them off … but I have control over how I react and respond to them” [9, interview 3]), decreased feelings of stigma, and decreased distress associated with unusual experiences after participating in the group^2^. Self-compassion emerged as a key theme. One Veteran stated, “The big thing is learning to be a bit kinder to myself when I am hearing stuff. I’m not so freaked out about it, and I’m a little bit kinder to myself when it does happen” [29, interview 2]. Another Veteran deepened his understanding of his voices and visions as motherly figures, linking them to past trauma and invalidation from his own mother in childhood – “A lot of my hallucinations have been older White women being kind and gentle and nurturing and trying to be helpful. So, I think that, in my darkest times, when I feel the worst … my brain is sort of manifesting some help for me” [25, interview 3]. Other Veterans also began acknowledging the complexity of their experiences, such as questioning whether voices really knew best, or realizing that voices may offer both good and bad advice. Finally, some Veterans appeared to shift their relationship with voices – e.g., approaching them with curiosity and respect rather than fighting against them, or viewing them as “just talk” with no real power.

In terms of decreased stigma, one Veteran reported that being in the group had “lowered the fear of what I think people are gonna say about me,” and led him to wonder whether others outside the group may be more supportive than he initially assumed [24, interview 2]. Another Veteran shared that the group helped him become more understanding of others’ struggles with mental illness, highlighting the potential benefits of the group in increasing empathy and reducing both self-stigma and stigma toward others. A third Veteran shared the groups “make me feel a bit more normal. It’s brought me comfort that I’m not alone. That’s the biggest thing” [15, interview 2].

Others began to consider their experiences from a more positive perspective (e.g., viewing experiences as a “special power” and not necessarily pathological). One Veteran mentioned enjoying discussions about cultural differences in mental health, and how unusual experiences may not always be seen as an “affliction” in certain cultures [25, interview 3]. Finally, some Veterans reported that it was helpful to engage in reality checking with other group members, or to be reminded that their experiences were attributable to mental health concerns, trauma, or other life events. For these Veterans, exposure to other group members’ perspectives helped shape their own understanding of unusual experiences in beneficial ways. One Veteran even shared that being in the group helped reinforce and deepen her appreciation for her voices – “I thought all voices were friendly, but I found out that they’re not. And I’m so glad mine are” [21, interview 2].

In contrast with Hornstein et al. (24)’s model, however, a few Veterans also did not report changes in understanding and/or coping with unusual experiences. Whereas Hornstein et al. (24) suggested that the ‘reframing’ phase would be associated with increased flexibility in thinking about unusual experiences and enhanced coping, one Veteran appeared to become more convinced of his framework. This Veteran endorsed beliefs in “telepathy” as well as the possibility that “I may replace certain sounds from outside and replace the noise with other noises, like voices” at the pre-interview [2, interview 1]. By the 8-week interview, the Veteran reported “I know what I have,” endorsing telepathy as the only explanation for his voices. Interestingly, one Veteran who did not endorse overall changes in unusual experiences was observed as endorsing certain experiences (suspicious ideas; believing thoughts could be heard by others) at the start of the group, but none at 16 weeks. It is unclear if this change is due to the group, a shift in this Veteran’s perspective or memory, or potential discomfort in the 16-week interview.

For many, having the opportunity to openly explore and consider various frameworks of understanding represented a new and transformative experience in and of itself. Rather than trying to avoid, control, or get rid of uncomfortable or confusing experiences, Veterans may instead be encouraged to approach these experiences with a sense of curiosity and self-compassion. In this way, Veterans may notice patterns or unique features of their experiences that lead to increased insight and meaning making.

As emphasized in our revised model, we observed a notable shift among Veterans in this study: instead of trying to eliminate, avoid, or suppress voices and other unusual experiences – or staying silent about them – participants gradually moved toward greater acceptance, curiosity, self-compassion, and a willingness to discuss these experiences and consider what they might mean (e.g., through questioning the origin, meanings, and cultural and contextual factors associated with these experiences and examining them through a more “analytical” lens). Most participants had previously been told their experiences were meaningless, “not real,” and revealed something wrong with them. For many, the group space represented a new opportunity to share and explore experiences that they had previously considered shameful, frightening, and indicative of pathology. The group’s connection, camaraderie, and acceptance of each member, along with seeing others in the group live and often thrive in different ways with their unusual experiences, seemed to encourage individuals to accept and explore their own experiences. One Veteran described this phenomenon:

“I haven’t had an experience with any medical professional talking about the specificities of what’s happening. Usually, they talk about these things in general terms, you’re seeing things, you’re hearing things, so we have to get you these medications. But it’s different to talk about what’s actually happening, what we’re actually seeing and hearing, and what it might mean to us. And hear about other people’s experiences. It’s just more human to me. I feel like a part of the conversation, and we’re all working through these things together and seeing what we can do. It’s different because a lot of times, medical professionals can make you feel like something’s wrong with you.” [25, interview 3]

In line with the idea of acceptance, some Veterans also spoke of allowing experiences to exist while redirecting attention and energy toward other goals: “I don’t really hope for them to go away, but I just hope to be able to live with them. Like, you’re not going to try to mess up my day, and I’m not going to try to mess up yours” [30, interview 3]. The positive coping strategies modeled by other group members appeared to reinforce these ideas, providing hope for living with these experiences rather than merely surviving them or waiting for a ‘cure’ to eliminate them.

For some Veterans, the process of acceptance manifested in changes in their beliefs about their own unusual experiences and ways of responding to them. Some Veterans made connections between their unusual experiences and adverse events they had lived through. Others realized their experiences were nuanced, had beneficial as well as detrimental aspects, and served needs for companionship or protection during difficult times. Others found a supportive space to share their own non-biomedical explanatory frameworks without being corrected or otherwise invalidated.

Finally, even Veterans who did not explicitly reframe or change their beliefs about their experiences were nonetheless still able to discuss and explore them in new ways within the group, leading to new insights and understandings.

### Facilitators and barriers to acceptance

The process of acceptance was facilitated through the validation of experience-based expertise and of explanatory frameworks beyond the illness model. Many Veterans commented that these frameworks were invalidated in their clinical care, but within the context of the groups, validating and paying attention to the personal meanings and interpretations of their experiences could be empowering, creating the possibility for new interpretations and coping strategies.

When participants shared their experiences, other group members often pointed out previously unrecognized strengths and forms of resilience. This type of supportive feedback tended to encourage further sharing and exploration within the group and reinforced the idea that one can have unusual experiences and still live a fulfilling life.

Barriers to acceptance included a persistent fear of engaging in one’s own or others’ unusual experiences – whether through reflection, exploration, or sharing – due to concerns that such engagement could worsen feelings of discomfort or distress. Some participants had been taught, often through prior treatment experiences or societal messaging, that the only appropriate response to unusual experiences was to eliminate or avoid them. Several participants expressed a preference for coping through avoidance or distraction, often because their experiences felt too noxious, malignant, or evil to try to understand or accept. For example, one participant described his voice as coming from Satan, and expressed that he would never turn toward Satan or try to understand his words [17, interview 2]. Others simply felt they already had an adequate understanding of their experiences and saw no value in exploring them further.

### From change to social integration

The third phase of the Hornstein et al. (24) model is Change. Following the validation and normalization of their experiences in Discovery and initial reductions in severity and/or distress of voices from Reframing, many voice hearers then experience greater confidence and self-efficacy. This leads to making changes within and outside the group, including strengthening social relationships and pursuing housing, education, or career-related goals. Hornstein et al. (24) further describe individuals who report reduced frequency of hospitalizations, reduced antipsychotic medication dosage, and improved sleep. Importantly, the authors point to lifestyle and symptom outcomes that vary according to an individual’s needs.

Among the Veterans in the current study, the most reported changes were 1) the adoption of new strategies for coping with voices and 2) increased social activity or desire for social connection. Nine Veterans reported utilizing new coping strategies following the group, including three Veterans who explicitly reported accepting their voices and two who reported various additional strategies (i.e., writing down what the voices say, talking back to voices, listening to music). One Veteran shared that while his coping strategy of listening to music remained the same, he now used it more intentionally – actively redirecting his attention with music rather than simply using it to avoid or drown out voices [29, interview 2]. Another Veteran shared that he started seeing an individual therapist, and that the combination of individual therapy and groups greatly enhanced his overall coping [30, interview 3]. Two Veterans mentioned reductions in medication dosage.

Additionally, nine Veterans endorsed changes in their social interest and behavior. Eight Veterans reported discussing their unusual experiences with friends or family members, which can serve to increase closeness as well as meaningful support outside of group members and treatment providers. Another Veteran reported sharing about unusual experiences with someone from his church (“It was like relief and release and just like, a burden was taken off me”) and connecting with another group member who also identified as Christian [6, interview 3]. This same Veteran reported that participating in the group helped him reflect on his social relationships more broadly: “I’m also realizing that some people that I’ve been hanging out with … don’t have my best interest at heart.” Other Veterans noted improved relationships with friends and family members due to enhanced communication skills, confidence, and self-awareness, reconnection with core values, and the ability to use the group space as an outlet for stress. Other social changes endorsed by Veterans included interest in meeting group members in person, desire to join a social or professional club, organizing community activities, and one Veteran who began a romantic relationship.

Other domains of change endorsed by multiple Veterans were in spirituality and personal goals and responsibilities. Two Veterans reported a desire to attend church, and one Veteran began attending following the group. One Veteran mentioned that he had enrolled in school and was taking classes to become a mechanic, stating that being in the group had helped him to “not quit yet” [26, interview 3]. Four Veterans endorsed increased motivation to spend more time in public spaces (“I kind of force myself out of the house”) [24, interview 3]. One Veteran, who initially struggled with feelings of suspiciousness and fear of leaving the house due to voices, shared that he had started using an evidence-checking strategy learned from other group members: “I look back on when I used to go out in the community, you know? How many times, when and where did I hear them? And nothing happened to me thus far, so nine times out of ten, ain’t nothing gonna happen … As time went on now, I’ve learned to realize [the voices] are not real” [18, interview 2].

Our refined model proposes that these changes – whether internal (e.g., increased feelings of agency, self-efficacy, or desire for social connection) or behavioral (e.g., spending more time with others or getting involved in community activities) – represent a broader process of social integration, where participants begin to explore and adopt new ways to claim their space in society, incorporating the insights and experiences gained from the group. We also found that six Veterans did not endorse any significant life changes as described in Hornstein et al. (24)’s Change phase.

### Facilitators and barriers to social integration

For Veterans in this study, facilitators to social integration included increased feelings of empowerment, motivation, and confidence to take social risks and expand social interactions. Identifying with others in the group – e.g., recognizing aspects of one’s own experiences in fellow group members’ stories – and hearing about others taking similar risks outside of the group appeared to reinforce participants’ own efforts to pursue social changes.

Barriers to social integration included feeling confined or limited by one’s unusual experiences and perceiving oneself as incapable of taking risks or initiating change – beliefs that may have been influenced by ongoing feelings of internalized stigma. Fear of distress and potential rejection also appeared to limit some participants’ willingness to engage socially. Some Veterans also expressed contentment or resignation with their current life circumstances, which may have contributed to reduced motivation to pursue new social connections or explore different roles within their communities.

## Discussion

This study makes several important contributions to the literature on the Hearing Voices approach by implementing an HVG-inspired group within a large, US-based public health setting, by using a longitudinal, repeat interview study design that begins with participants who are new to HV, and by providing a model of change for the first few months of engagement with groups. Veteran experiences in VVV groups largely aligned with Hornstein et al. (24)’s three-phase model of transformation but also supported a refined model highlighting the processes of Connection, Acceptance, and Social Integration. Veterans commonly described feeling less alone, more self-compassionate, and more accepting of voices and other unusual experiences, as well as increased coping and social engagement due to group participation. These processes were facilitated by feeling validated and supported by peers, shared identity with other Veterans, and nonjudgmental, open discussion, while mistrust, perceived invalidation, unequal participation, avoidance, stigma, and fear of rejection were some of the observed barriers to change. In the following sections we consider aspects of our study with respect to Hornstein et al. (24) and the broader literature on HVGs.

### Setting

While much of the existing literature on HVGs is on community-based groups within HV networks and outside of clinical spaces (23, 38), there is an emerging literature on HVGs within formal mental healthcare settings, though largely outside of the US (27, 31, 32, 39). This study contributes to the literature on HVGs within clinical systems from the vantage point of a US-based public healthcare system. Regarding our study question comparing the Hornstein et al. (24) model from civilian, largely in-person HVGs to virtual VVV groups in the VA, our findings indicate that these groups can be successfully adapted to this context in ways that maintain the core characteristics of HV groups based in the community.

While the VA is a biomedically oriented system, it does endorse a recovery approach and there are additional recovery-oriented services Veterans are able to access. VVV complements these services while offering a unique meaning-oriented, participant-driven approach to psychosis. Prior research has found that HV groups led by clinicians tended to be more structured which, while not inherently positive or negative, influences certain group processes such as information sharing and self-reflection (40). As the non-hierarchical, unstructured nature of HVGs is thought to be fundamental in promoting participant autonomy and empowerment (24), future research should continue exploring group facilitation approaches that uphold these values, particularly when clinicians are involved in cofacilitation.

### Demographics

Although both studies took place in the United States and involved groups grounded in Hearing Voices Network-USA charter values and principles, there were important demographic and diagnostic differences between the populations studied. Hornstein et al. (24) sampled and interviewed a subset of community participants from across the US, where 56% of participants were diagnosed with psychotic disorders and most identified as White (66%). In contrast, this study was conducted within a single integrated healthcare system and included repeat interviews of group participants who were all diagnosed with psychotic disorders, with 50% identifying as Black. Additionally, all of our participants were Veterans, a group whose shared military service and service-related disability status distinguishes them from the general population in significant ways, including a unique group camaraderie based on shared military culture and adversities and access to resources from the VA. These differences likely reflect distinct lived experiences and social contexts that may account for some of the variation in our findings and models of change. While our pre-intervention interviews did indicate our participants held a broad range of explanatory frameworks regarding their experiences coming into the group, it could be that they also had internalized biomedical understandings to a greater extent than Hornstein et al. (24)’s sample, making it even more remarkable that their model aligned with our findings, but perhaps constraining the degree of reframing that was possible in the brief time span of the study.

Recruitment strategies differed between the two studies, which also influenced the sample characteristics. Hornstein et al. (24) relied on self-referral methods, which likely attracted individuals already positively and consistently engaged with the HV movement and various HV groups. In contrast, our participants were recruited through clinical providers within the VA, and all attended the same set of groups. Our study likely captured a broader range of participants, including regular VA service users who had not previously heard of HVM. Our sample also included those who attended infrequently and who may have had less favorable experiences, offering valuable insights into early facilitators and barriers to engagement and transformation.

### Design

While much of the literature consists of cross-sectional studies and surveys (23, 38), this study takes a longitudinal approach with repeat interviews prior to group engagement and then after 8 and 16 weeks. This provides insight into within-person processes of change and gives our model additional depth and dimension. Our model pays attention to internal individual processes as well as relational processes within the group and outside the group.

A key difference between this study and Hornstein et al. (24) was the amount of time study participants had been involved in their respective groups. In Hornstein et al. (24), 57% of participants were in a group for over a year and 23% for more than three years. In our sample, attendance ranged from a single group session to 16 sessions (4 months), with a median of 10 sessions (2.5 months). This may underlie some of the differences in our data that led us to refine the model to better reflect the experiences of our study participants (**Figure 1**). Our refined model of change may represent early engagement experiences with the HV approach. Longer-term group involvement likely allows for more profound changes, particularly in the later phases of the models. Many of the Veterans who reported no major life changes did report reduced feelings of isolation and shifts in their thoughts about their voices, suggesting that these individuals made gains in the Connection and Acceptance phases, and may have endorsed additional changes with time.

Many aspects of our model have been reported in the literature on HV. Connection and reduced isolation are common themes (38, 40–42). Acceptance of voices and of oneself as a voice hearer is also a key value of the HVM (20, 39, 43–45). Social integration is perhaps the most difficult of these factors to assess, but a sense of connectedness that extends beyond the group itself has been reported in the literature (26, 46, 47).

### Limitations

The short-term nature of the VVV group study may have limited the degree of changes we were able to observe, particularly in the realms of self-understanding, reframing, and relating with others. While studying longer-term groups may offer more opportunities for sustained growth and transformation, the 16-week timeframe was chosen for feasibility within the study timeline and alignment with other evidence-based group psychotherapies offered by the VA and similar health systems. A follow-up study with these same participants might provide insights into changes that occur over longer periods.

All the groups in this study were virtual, a condition initially imposed on the cohort by the COVID-19 pandemic, but which many Veterans felt facilitated their participation. It is possible that the virtual format affected group composition, however, as while it may have included certain individuals, it likely excluded others. The virtual format also could affect group dynamics, and most research on HVGs likely has been on in-person groups, though there is an emerging literature on virtual groups as the format has become more common (48, 49).

The VA is a unique population and context, as Veterans share a camaraderie around their shared military background and have access to more resources within the VA Healthcare System than is typically available in non-VA public health systems in the United States. At the same time, Veterans experience significantly higher rates of mental health disorders compared to the general population, driven by unique military-related exposures and the challenges of transitioning to civilian life (50). Prevalence of schizophrenia, the prototypical psychotic disorder, is particularly high among US Veterans (51). Veterans are predominantly male, as is reflected in our sample (52). Half of our sample also identified as Black, reflecting the demographics of this health system. All these factors may influence the transferability of our findings to other populations and healthcare settings.

VVV was a novel intervention at VA, and most of our facilitators were newly trained and had never facilitated an HV group before. This could also have affected our outcomes, as expert, experienced facilitators may have achieved different outcomes, but the fact that novice facilitators could still produce these outcomes bodes well for our ability to offer groups that approximate HV dynamics.

### Conclusion and future directions

This study examined the impact of a Hearing Voices-inspired group approach, Veteran Voices and Visions (VVV) group, on Veteran participants’ perspectives and life experiences. We used the three-phase model of transformation proposed by Hornstein et al. (24) as the point of departure for our analysis. While we found many areas of alignment with their model, we also observed some interesting differences that led us to refine the model to better reflect the unique experiences of our study participants and the context in which the VVV groups were delivered.

For most participants, the groups provided a valuable setting for connection, acceptance, and community that led to a greater sense of belonging and social integration. Participants were encouraged to explore multiple, sometimes opposing, beliefs about their unusual experiences with curiosity and without judgment, in the face of important cultural and contextual factors. In this way, the group process helped normalize and validate these experiences as meaningful and worthy of understanding on their own terms, rather than viewing them solely as symptoms to be eliminated. As a result, many Veterans described increased self-compassion and even respect for these experiences, as well as an increased willingness to examine and engage with them.

Overall, our findings show that the process of transformation can take many different forms, rather than being defined by a single marker of success, emphasizing the importance of a person-centered approach to recovery. As groups are participant-driven and Veterans enter with varying needs and expectations, it is likely that not everyone will get exactly what they want out of every group session. This does not indicate a failure of the group or individual but rather reflects the diverse goals and perspectives of the participants, which can shape the group experience in unique ways. By making space for the complexity of lived experience, VVV groups can support personalized pathways toward recovery, or toward living meaningfully with voices and other unusual experiences. The HV and VVV ethos encourage group engagement without predefined behavioral targets or outcomes, allowing for individualized experiences. As each Veteran may gain something different from the group, non-conformity to the three-phase model does not necessarily imply a lack of meaningful benefit. Our framework accounts for diverse trajectories of growth, including the possibility that additional life changes may emerge over time and that social integration can take many forms and may unfold gradually or internally. Future research should consider exploring these alternative outcomes with the understanding that an individual’s way of relating and responding to their unusual experiences may be continually evolving.

Our findings also suggest that the VVV group model may be adapted to other healthcare contexts, including other VA systems and settings serving Veterans and individuals with serious mental illness. Further research is needed to determine how best to integrate these approaches into formal health systems (53). Future research should also explore the longer-term effects of VVV groups to better understand their potential for fostering change.

## Data Availability

The datasets presented in this article are not readily available because they cannot be fully anonymized. Requests to access the datasets should be directed to the corresponding author.

## Footnotes

1 Study participant number and interview number (in that order) are provided after a participant’s comments or experiences are described.

2 This analysis is based on qualitative interview data, though our other study analyzing quantitative data including this sample found that group participation led to a statistically significant reduction in distress and increased benevolence associated with voices, as measured by two separate rating scales (see 34).

## Appendix 1: Qualitative Interview Guide for weeks 6 and 12

1. Tell me about your experiences in the Veteran Voices and Visions group. What is it like going to the group?

2. How is the Veteran Voices and Visions group different from other mental health services you have used?

3. What do you like about the group?

4. Which experiences have you had in the group which most stick in your head?

5. How has your understanding of your voice hearing (or any other) experiences changed since attending the group? How has the group changed this?

6. Has your ability to live with your voices changed as a result of your participation in the group? If so, how?

7. How has it been/what has it been like to get to know the other Veterans in the group?

8. Have relationships with people outside the group changed at all for you since you started the group? Has your experience in the group played a role in this at all?

9. What are your hopes for your (voice-hearing) experiences in the future?

10. How would you describe any changes in your life (if any) because of going to the groups? How did going to the group bring about this change?

11. Is there anything you dislike about the group?

12. Why have you kept/stopped attending? What might keep/stop you from coming to the group?

13. How was it participating in the group over video conference?

  a. If you had the choice of an in-person vs. a virtual group, which would you choose?

14. What did you find most important about attending the group?

15. What would you recommend to us as we go forward with the approach?

16. Is there anything we have not talked about that we should know to better understand what VVV is all about?
